# Experimental and survey-based data on willingness to pay for seafood safety and environmental sustainability certification in Nigeria

**DOI:** 10.1016/j.dib.2020.105540

**Published:** 2020-04-14

**Authors:** Kelvin Mashisia Shikuku, Nhuong Tran, Lauren Pincus, Vivian Hoffmann, Carl Johan Lagerkvist, Shehu Latunji Akintola, Kafayat Adetoun Fakoya, Jacquieline Muliro

**Affiliations:** a WorldFish, Jalan Batu Maung, Batu Maung 119, Penang Malaysia; b WorldFish, Yangon, Myanmar; c International Food Policy Research Institute, Nairobi Kenya; d Swedish University of Agricultural Sciences, Uppsala Sweden; e Lagos State University, Lagos Nigeria

**Keywords:** Aquaculture, fish value chain, seafood safety, certification, willingness to pay, Nigeria

## Abstract

Aquatic foods, including fish, are a substantial component of animal source foods globally, and make a critical nutritional contribution to diets in many contexts. In the global North, concern among consumers and regulators over the safety and environmental sustainability of seafood, particularly in developed nations, has led to the development of increasingly stringent seafood safety standards. While such standards may constitute regularity, logistical, and economic barriers to participation in export markets by small-scale producers, they have in other contexts catalysed upgrades to production and post-harvest handling practices within value chains associated with both capture fisheries and aquaculture. The health burden of foodborne illnesses is a major concern in developing countries. As incomes rise, consumers in developing countries are increasingly willing to pay a premium for safer and environmentally sustainable foods. However, there is little empirical evidence on consumers’ willingness to pay for seafood safety in developing countries, particularly in sub-Saharan Africa. Data on demand for seafood safety and environmental sustainability certification in African countries are largely unavailable in the public domain. In this paper, we describe data collected in Lagos State, Nigeria in October and November 2019. Experiments in the form of Becker-DeGroote-Marschak (BDM) auction mechanism, and post experiment surveys were conducted with 200 fish consumers in fish markets. These data can be used to assess whether consumers’ demand for safe and healthy seafood from local markets can be harnessed to generate positive economic returns to producers.

Specifications Table

**Table:** 

Subject	Social Sciences
Specific subject area	Food safety, Aquaculture certification
Type of data	Excel (csv) files
How data were acquired	A framed experiment and survey
Data format	Cleaned raw data
Parameters for data collection	Data were collected from randomly selected individual fish consumers. Every second potential buyer of fish was recruited for participation. Experiment participants bid on eight fish products.
Description of data collection	A framed experiment in the form of Becker-GeGroote-Marschak (BDM) auction mechanism was conducted at the point of fish purchase. A post-experiment survey was then conducted to collect background information. Experimental data were captured using recording sheets and later digitized in open-data-kit (ODK). These data contain information on the bids submitted during the experiment and measuring consumers’ willingness to pay for food safety and environmental sustainability certified fish. Survey data were gathered using paper questionnaires and later digitized in ODK. These data contain information about demographic characteristics of the consumers, consumption and expenditure patterns, knowledge and perceptions, preferences for brand purchases, and trust.
Data source location	City/Town/Region: Lagos State Country: Nigeria
Data accessibility	Available at: https://doi.org/10.7910/DVN/SXQKZ0

## 1 Value of the data

• The data can allow researchers to understand whether consumers are willing to pay a price premium for seafood safety, what types of consumers are willing to pay, and whether the premium is high enough to incentivize producers to adopt aquaculture best management practices;
• The data also allow researchers to control for additional covariates in the analysis of willingness to pay for aquaculture certification thereby increasing the precision with which consumer demand is measured and providing means to explain important determinants of willingness to pay;
• Furthermore, because the data capture willingness to pay for different fish products varying by size, form, and price, it provides an opportunity for researchers to assess whether the value of aquaculture certification schemes is symmetric or asymmetric.

## 2 Data description

This paper presents data collected in October and November 2019 to assess consumers’ demand for seafood safety certification. Experimental data contains information about a warm up market exercise that was conducted using sweets in order to ensure participants’ full understanding of the market exercise. The data also contain information about three control questions that were asked to assess the participants’ understanding. Most importantly, the data contains the bids submitted by consumers for eight fish products. The post-experiment survey questionnaire was divided into 6 modules. Module A focused on the geographical location of the participant. Module B collected data about demographic characteristics of the consumers. In Module C, asked consumers questions about consumption and expenditure on fish and other food items. Module D focused on knowledge and perceptions about food safety. In Module E, we asked consumers about their preferences for brand purchases. Lastly, in Module F, data on consumers’ trust in local market inspectors and government inspectors of food safety were collected.

## 3 Experimental design, materials, and methods

### 3.1 Experimental design

An economic field experiment in the form of the Becker-DeGroote-Marschak [1]—henceforth, BDM—mechanism was conducted to elicit consumers’ willingness to pay (WTP) for safety and environmental sustainability certified fish. In the BDM, individuals compete against a random draw or bid from a known distribution; if the submitted bid beats the random draw, the individual purchases the product and pays a price equal to the random draw; if the bid is lower, on the other hand, the individual does not purchase.

The data were collected with the objective to measure the premium consumers are willing to pay for safety and environmental sustainability certified fish. The experiment was conducted with a random sample of 200 consumers. The experiment involved eight (8) catfish products. Catfish is the most commonly produced and consumed fish in Nigeria. The products varied in certification status (certified/safety labelled vs non-certified/non-safety labelled), size (250 g vs 500 g for smoked fish; and 500 g vs 1000 g for live fish), and form (e.g., smoked and live).

Certified fish was obtained from producers approved for following guidelines and criteria developed by the government in their production activities. A list of certified producers in Lagos state was obtained from the government. This was followed by a validation exercise to confirm that farmers had proof of certification by the government. The certified producers were then contacted to collect information about the species they produced, the different forms in which the products are sold, the main buyers, the typical sizes sold, the prices charged, and whether fish would be available for selling to us during the experiment period. Non-certified fish were obtained from the local market. All original packaging information was removed from the products prior to the experiment to control for any reaction individuals might have to the packaging. Smoked fish was displayed in clear freezer bags. For live fish, the containers in which the fish are kept before selling were labelled instead.

Upon consenting, a participant received an endowment of 1500 Naira cash token as the show-up fee. The show-up fee in our experiment is twice the price of 1 kg live catfish. The enumerator then described each product—one at a time. For the certified products, the information contained in the label and explaining the method that was used to produce the fish, was read out aloud. No additional information was provided. For the non-certified fish, the consumer was informed that the method that was followed in production was unknown. After describing the products and before the bidding begins, the enumerator displayed each of the products on the table. To prevent potential order effects, the order in which the products were displayed was randomized. Randomization was done prior to the experiment and the randomized order given to enumerators. The participant was allowed to ask any question related to the fish products or the experiment.

The participant was then informed that a simple market exercise would be used to give him or her a chance to buy fish products for him/herself and family members. He/she was told that depending on his/her choices, and also random chance, he/she may actually purchase one of the seafood products offered through this procedure, or he/she may not. The participant was encouraged to listen attentively to understand exactly how the market exercise would work. The enumerator explained that he/she was part of a research team, not a sales person for any particular type of fish; but, as part of this exercise he/she (i.e. the enumerator) may sell the participant some fish.

The real experiment with fish was preceded with a warm-up exercise. The warm-up demonstrated the market exercise using candies. To ensure that instructions have been clearly understood, the participant was asked three basic questions related to the experiment. The enumerator only proceeded when all the three were answered correctly.

Although an individual bid for eight fish products, only one was actually purchased if the bidding price exceeded the reservation price for that product. Selection of the product was determined randomly in STATA prior to the experiment.

### 3.2 Sampling design

Lagos state was purposively selected because of the considerable number of certified aquaculture producers, currently targeting export markets. Consumers were recruited at three types of outlets for fish: main fish markets, roadside markets, and supermarkets. The number of each outlet to include was informed by key informant interviews and focus group discussions. We conducted a field experiment at the points of purchase of fish [2]. No subject was allowed to participate in more than one treatment. In all selected outlets, each second consumer was approached, before making his/her intended purchase, and asked to participate in the experiment. Upon consenting the participant was led to a quiet tent within the market where the experiment was conducted. The experiments was conducted in November 2019 by enumerators specially hired and trained, and supervised by professors from Lagos state university, in collaboration with WorldFish. The final sample comprised 200. Prior to the actual experiment, piloting was done. A brief survey was conducted at the end of the experiment.

### 3.3 Data management and code availability

During data collection, the corresponding author checked for inconsistencies, patterns and outliers in the data, and provided feedback to the field team each day whenever necessary. Experimental data were collected using pre-designed recording sheets while survey data were captured using a paper questionnaire. All data were then digitized in open-data-kit (ODK) and submitted online to WorldFish. Data were then imported into STATA for cleaning. The do files (Nigeria_WTP_experiment1.do and Nigeria_WTP_postexperiment1_survey.do) that were used to clean raw data have been shared online at: https://doi.org/10.7910/DVN/SXQKZ0.

## Appendix Supplementary materials

Image, application 1
